# Efficient in vivo bone formation by *BMP-2* engineered human mesenchymal stem cells encapsulated in a projection stereolithographically fabricated hydrogel scaffold

**DOI:** 10.1186/s13287-019-1350-6

**Published:** 2019-08-14

**Authors:** Hang Lin, Ying Tang, Thomas P. Lozito, Nicholas Oyster, Bing Wang, Rocky S. Tuan

**Affiliations:** 1 Department of Orthopaedic Surgery, Center for Cellular and Molecular Engineering, University of Pittsburgh School of Medicine, 450 Technology Drive, Pittsburgh, PA 15219 USA; 2 Molecular Therapeutics Laboratory, Department of Orthopaedic Surgery, University of Pittsburgh School of Medicine, 450 Technology Drive, Pittsburgh, PA 15219 USA; 3 McGowan Institute for Regenerative Medicine, University of Pittsburgh School of Medicine, Pittsburgh, USA; 4 Department of Bioengineering, University of Pittsburgh Swanson School of Engineering, Pittsburgh, PA USA; 5 Institute for Tissue Engineering and Regenerative Medicine, The Chinese University of Hong Kong, Hong Kong SAR, China; 6 Present Address: Center for Pulmonary Vascular Biology and Medicine, Department of Medicine, University of Pittsburgh School of Medicine, Pittsburgh, PA USA; 7 Present Address: Department of Orthopaedic Surgery, Keck School of Medicine, University of Southern California, Los Angeles, CA USA

**Keywords:** Osteogenesis, Bone tissue engineering, Bone formation, 3D bioprinting, Gene therapy, Ex vivo gene transduction

## Abstract

**Background:**

Stem cell-based bone tissue engineering shows promise for bone repair but faces some challenges, such as insufficient osteogenesis and limited architecture flexibility of the cell-delivery scaffold.

**Methods:**

In this study, we first used lentiviral constructs to transduce ex vivo human bone marrow-derived stem cells with human bone morphogenetic protein-2 (*BMP-2*) gene (*BMP*-hBMSCs). We then introduced these cells into a hydrogel scaffold using an advanced visible light-based projection stereolithography (VL-PSL) technology, which is compatible with concomitant cell encapsulation and amenable to computer-aided architectural design, to fabricate scaffolds fitting local physical and structural variations in different bones and defects.

**Results:**

The results showed that the *BMP*-hBMSCs encapsulated within the scaffolds had high viability with sustained *BMP-2* gene expression and differentiated toward an osteogenic lineage without the supplement of additional BMP-2 protein. In vivo bone formation efficacy was further assessed using an intramuscular implantation model in severe combined immunodeficiency (SCID) mice. Microcomputed tomography (micro-CT) imaging indicated rapid bone formation by the *BMP*-hBMSC-laden constructs as early as 14 days post-implantation. Histological examination revealed a mature trabecular bone structure with considerable vascularization. Through tracking of the implanted cells, we also found that *BMP*-hBMSC were directly involved in the new bone formation.

**Conclusions:**

The robust, self-driven osteogenic capability and computer-designed architecture of the construct developed in this study should have potential applications for customized clinical repair of large bone defects or non-unions.

**Electronic supplementary material:**

The online version of this article (10.1186/s13287-019-1350-6) contains supplementary material, which is available to authorized users.

## Background

Large bone defects, fracture-delayed unions, and non-unions constitute 10–15% of bone fractures reported in the USA annually [1] and are normally not repaired by the body, thus representing a major clinical challenge. For example, scaphoid and osteoporotic fractures, which are common in the elderly population, are difficult to completely heal and are often accompanied by pain or prolonged hospitalization [2, 3]. Autologous bone graft procedures have been considered as the “gold standard” for nonunion fractures [4, 5], which however cause donor site morbidity and are greatly restricted by tissue availability [6].

Research advances in tissue engineering, involving a combination of cells, biomaterial scaffolds, and signaling molecules [7–9], have shown its potential in enhancing bone healing without the use of native bone tissue [10, 11]. Owing to their osteogenic capability upon stimulation, relative ease of isolation, low immunogenicity, and lack of ethical controversial [12], mesenchymal stem cells (MSCs) isolated from various adult tissues have been tested in different animal models as a promising cell type for bone repair, including femoral defects [13, 14], mandibular defects [15–17], and tibial defects [18, 19]. Scientific consensus indicates that efficient osteogenesis of MSCs requires the sustained stimulation by osteo-inductive biofactors, such as bone morphogenetic protein-2 (BMP-2) [20]. BMP-2 is a member of the transforming growth factor-β superfamily and promotes bone formation by directing MSC differentiation into osteoblasts/osteocytes, which produce the requisite extracellular matrix of bone tissues [21–23]. Human recombinant human BMP-2 (rhBMP-2) is currently approved by the US Food and Drug Administration (FDA) for clinical uses such as spine infusion. However, the application of BMP-2 protein still poses some challenges, such as its short half-life and rapid systemic clearance by the bloodstream in vivo [24], while successful bone formation requires long-term active osteoinduction or osteoconduction [25, 26]. To maintain a high concentration of BMP-2 for extended periods of time at the defect site, a large dose of BMP-2 protein is often applied at the beginning, which is costly and might cause side effects, such as edema, ectopic bone formation, and nerve root irritation [27].

Currently, there are two major ways to overcome this issue: (1) controlled release through physical binding/trapping of BMP-2 protein within the delivery vehicles [28] or (2) gene transfer-based genetic engineering of cells with the *BMP-2* gene [29]. For example, rhBMP-2 was encapsulated in poly (lactic-co-glycolic acid) (PLGA) microparticles, with a sustained release profile of over 5 weeks, to bridge a rat critical cranial defect [30]. A gelatin hydrogel has also been used to trap rhBMP-2, resulting in sustained release and uniform bone formation [31]. Controlled release technology, however, usually requires relatively complex material modifications or fabrication and may not be able to retain the full bioactivity of the BMP-2 released in the late period. In view of the benefits of viral vectors, including long-term, efficient gene transfer and relatively low cost [32], we and other research groups have introduced *BMP-2* gene into cells, through in vivo [33, 34] and ex vivo [35, 36] gene transfer, to accelerate bone healing. Compared to BMP-2 protein delivery, ex vivo gene transfer approach has been proved to be more efficient in maintaining the BMP-2 concentration and bioactivity without the need of a large initial dose, with the potential of promoting faster and more efficient bone formation at the defect site.

To deliver and localize growth factors into large bone defects and nonunion sites, scaffolds are often utilized as the carrier and as the support for local tissue regeneration. Ideal scaffolds not only support cell growth and differentiation, but also have functionality as a template for effective bone healing [9, 37]. Because of the physical and structural variations in different bones and defects, regenerative scaffolds must offer flexible design capabilities to replicate or compensate for the local tissue anatomy. To meet this requirement, one method is to employ medical imaging of the lesion and surrounding tissues or three-dimensional (3D) models as a design template for bone scaffold fabrication. We recently developed a live-cell fabrication technology using visible-light-based projection stereolithography (VL-PSL), which was capable of producing solid or porous scaffolds with desired architecture [38]. In addition, the procedure allowed live cells to be incorporated in to the monomer solution and thus encapsulated within the engineered scaffolds during the fabrication procedure, without an additional cell-seeding step. The cells were uniformly distributed throughout the scaffolds, instead of being located primarily on the scaffold surface, and should thus promote more complete tissue repair. However, the poly (ethylene glycol) diacrylate (PEGDA) used was not readily biodegradable, and the naïve MSCs showed limited bone formation ability without sustained osteogenic stimulation [39, 40].

**Fig. 1:**
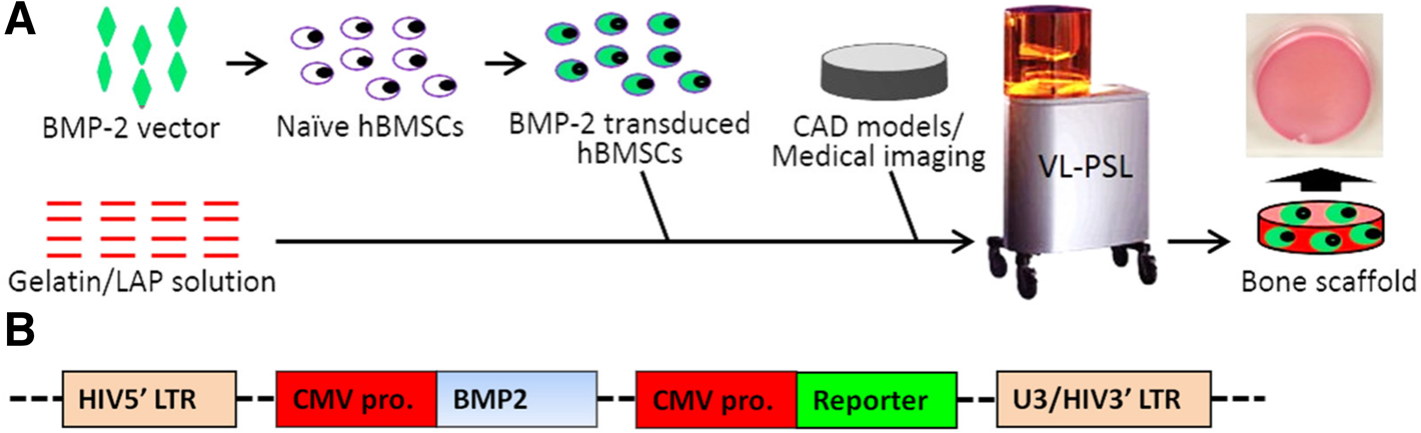
**a** Fabrication scheme of bone scaffold using VL-PLS, with simultaneous incorporation of lenti-*BMP-2* vector-transduced hBMSCs. **b** Structure of the *BMP-2* lentiviral construct used in this study. CMV pro., cytomegalovirus promoter; HIV, human immunodeficiency virus; LTR, long-terminal repeat. eGFP was used as the reporter

In this study, we fabricated bony constructs that possessed sustained and self-driven osteogenic capability, as well as a customized geometry and architecture. In this process, as schematized in Fig. 1a, human bone marrow-derived MSCs (hBMSCs) that had been transduced with a lenti-*BMP-2* construct were encapsulated within a gelatin-based scaffold using VL-PSL technology. Gelatin was employed here as the biomaterial because it is biocompatible and biodegradable, supplies cell-binding ligands, and supports bone formation [41–43]. We hypothesized that the ex vivo *BMP-2* gene-transduced hBMSCs could be uniformly distributed throughout the VL-PSL-fabricated scaffolds and would sustainably express *BMP-2* gene to drive hBMSC osteogenesis and efficient bone formation in vivo without the addition of exogenous BMP-2 protein. The extent of bone formation was estimated based on alkaline phosphatase (ALP) activity, gene expression analysis by real-time polymerase chain reaction (real-time PCR), osteocalcin immunohistochemistry (IHC), histological staining, and microcomputed tomography (micro-CT) imaging. Our findings showed rapid bone formation in vitro and in vivo by the bone constructs fabricated with designed geometry and architecture by this novel procedure, suggesting its potential application for clinical repair of large bone defects or non-union.

## Methods

All chemicals used in this study were purchased from Sigma-Aldrich (St. Louis, MO) unless otherwise stated.

### Preparation of materials for visible-light projection stereolithography (VL-PSL)

The photoinitiator lithium phenyl-2,4,6-trimethylbenzoyl phosphinate (LAP), with absorbance ranging from ultraviolet (210 nm) to visible (500 nm) light, was synthesized as described by Fairbanks et al. [44]. Methacrylated gelatin (mGL) was synthesized according to a procedure previously described with slight modification [45, 46]. Briefly, gelatin (15 g) was dissolved in 500 ml of deionized H_2_O at 37 °C, and 15 ml of methacrylic anhydride (MA) was then added. The mixture was placed in a 37 °C shaker at 110 rpm for 24 h and then dialyzed for 4 days against deionized H_2_O (2000 NMWCO dialysis tubing, Sigma-Aldrich). After lyophilization, the mGL sponge was stored in a desiccator protected from light. The methacrylation efficiency of mGL was ~ 80% [46].

### *BMP-2* gene transduction of human BMSCs (*BMP*-hBMSCs)

hBMSCs were isolated from human bone marrow obtained from the femoral heads of a patient (60-year-old male) undergoing total hip arthroplasty with Institutional Review Board approval (University of Washington and University of Pittsburgh). The hBMSC isolation and culture followed a standard procedure in our laboratory. hBMSCs were cultured in hBMSC growth medium (GM, α-MEM containing 10% fetal bovine serum (FBS, Invitrogen, Carlsbad, CA), 1% antibiotic-antimycotic, and 1.5 ng/ml FGF-2 (RayBiotech, Norcross, GA)). Once 70 to 80% confluence was reached, cells were passaged or cryopreserved. hBMSCs were validated as capable of colony formation and osteogenic, adipogenic, and chondrogenic differentiation upon stimulation (data not shown). All experiments were performed with passage 3 (P3) hBMSCs. As shown in Fig. 1b, a lentiviral construct carrying both human *BMP-2* and enhanced *green fluorescent protein (eGFP)* genes driven by human cytomegalovirus (CMV) immediate early enhancer and promoter was designed for the genetic engineering of hBMSCs. Lentiviral preparations were manufactured through four-plasmid DNA co-transfection system according to our published protocol [47]. The titer of the lentiviral preparation was estimated by limiting dilution to be around 5 × 10^6^ infectious units (i.u.)/ml of GFP reporter. We transduced hBMSCs with the BMP-2 lentiviral construct (multiplicity of infection 5) in the presence of Polybrene (8 μg/ml) for 10 h; the transduced cells were further culture-expanded. Forty-eight hours later, transduced cells were monitored to determine the transfer efficiency of *eGFP* in living hBMSCs under a fluorescence microscope.

### Bone scaffold fabrication using VL-PSL

Live-cell scaffold fabrication using VL-PSL was performed following our established, published protocol [38]. Briefly, mGL was dissolved in Hank’s balanced salt solution (HBSS, Invitrogen) at 10% (w/v), and the pH of the solution was adjusted to 7.4 using 10 N NaOH. While protected from light, LAP was added to an mGL solution at 0.6% (w/v) until dissolved completely. The final solution was referred to as PSL solution. Lentiviral *BMP-2*-transduced hBMSCs were pelleted and resuspended in PSL solution at a final density of 2 × 10^6^ cells/ml. The cell-laden PSL solution was immediately poured into the basement plate in the VL-PSL device, and live-cell gelatin scaffolds were produced using a computer-aided design (CAD)-derived 3D structure at default resolution **(**Additional file 1: Figure S1). The fabrication of scaffolds with 2 mm height took approximately 30 min. After VL-PSL, the constructs were washed in HBSS twice and then cultured in GM without FGF-2. This group was referred to as “Gene.” As control, naïve constructs encapsulating untransduced hBMSCs were fabricated using the same condition but included 100 ng/ml BMP-2 during the fabrication process. This group was referred to as “Protein.” The culture medium of both groups was changed at day 2 and day 7, and then every 3 days using GM without FGF-2 or BMP-2.

### BMP-2 activity tested by ELISA and alkaline phosphatase (ALP) staining

The BMP-2 protein in the medium of both the Protein and Gene groups at day 7 was quantified using an ELISA kit (R&D, Minneapolis, MN), following the manufacturer’s instructions. At day 14, ALP activity in both groups was analyzed by direct ALP staining with the Leukocyte Alkaline Phosphatase Kit (86R, Sigma) following the manufacturer’s instructions. Briefly, the cultured constructs were removed from the culture medium, immersed in the staining solution, and returned to the incubator for 1 h. The staining of whole constructs or cells was imaged using a DSL camera (T3i, Cannon, Japan) or a microscope (CKX41, Olympus, Japan) equipped with a color camera (DFC321, Leica, Germany).

### Real-time RT-PCR

Total RNA was extracted using TRIzol reagent (Invitrogen, Carlsbad, CA) following the standard protocol and purified with the RNeasy Plus Mini Kit (Qiagen, Hilden, Germany). A SuperScript III kit (Invitrogen, Carlsbad, CA) was utilized to complete the reverse transcription with random hexamer primer. Real-time RT-PCR was performed using a StepOnePlus thermocycler (Applied Biosystems, Foster City, CA) and SYBR Green Reaction Mix (Applied Biosystems). The expression of osteogenic marker genes, including osteocalcin (*OCN*) and bone sialoprotein II (*BSP II*), was analyzed. The 18S transcript level was used as an endogenous control, and gene expression fold changes were calculated using the comparative CT (ΔΔCT) method.

### Mechanical testing

Constructs from the Protein or the Gene groups at days 0 or 56 post-fabrication were tested under 10% uniaxial, unconfined compression in an electromechanical tester with a 250-g load cell (ElectroForce 3200, Bose, Eden Prairie, MN). The construct cylinders (5 mm D × 2 mm H) were placed between stainless steel disks and preloaded to 0.6 g. The samples were subjected to 10% strain applied at room temperature using a constant rate (0.05%/s). Compressive moduli of constructs were determined from the slope of force versus displacement plots.

### Immunohistochemistry (IHC)

After 28 days of culture, cells within the scaffolds were fixed in formalin (10% phosphate buffer, Fisher Scientific) and characterized by IHC for the expression of OCN. Heat-mediated antigen retrieval was performed with sodium citrate buffer (pH 6.0) for 20 min at 95 °C. Endogenous peroxidase was blocked with 3% H_2_O_2_ in methanol for 10 min, and nonspecific binding was suppressed with 1% horse serum in PBS for 45 min. Following antigen retrieval and blocking, the samples were incubated with rabbit anti-OCN primary antibody (1:200) (Abcam ab93876, Cambridge, MA) or an isotype control antibody (Abcam) overnight at 4 °C, followed by 30 min of incubation with horse anti-rabbit biotinylated secondary antibodies (Vector Laboratories, Burlingame, CA). Staining was developed by incubating the samples with horseradish peroxidase-conjugated streptavidin (Vector Laboratories) and treating the samples with the Vector® NovaRED™ peroxidase substrate (Vector Laboratories). Following IHC, the samples were counterstained with hematoxylin, dehydrated, mounted, and coverslipped, and images were captured with an Olympus CKX41 microscope (Center Valley, PA).

### Animal and scaffold intramuscular implantation

To evaluate in vivo bone formation, the bioactive scaffolds (the Protein or the Gene groups, 4 samples per group) were individually implanted intramuscularly in both hindlimbs in 2-month-old severe combined immunodeficiency (SCID)/J mice (Jackson Laboratory, Bar Harbor, ME) using an Institutional Animal Care and Use Committee (IACUC)-approved protocol (15014817). The experiment was conducted in conformity with the “Guiding Principles for Research Involving Animals and Human Beings” as adopted by The American Physiological Society. Mice were randomly assigned to each group. After 56 days, the mice were sacrificed, and samples were collected as follows: (a) One sample randomly from each group was fixed in formalin for histological analysis; (b) Three samples from each group were put in PBS for immediate mechanical testing and then placed in 1 N HCl for calcium quantitation.

In addition to assessment of bone formation, we also examined if the transplanted *BMP*-hBMSCs could directly contribute to the formation of bone tissues. Since only samples in Gene group could form bone, four constructs from this group were implanted intramuscularly. After 14 or 28 days, samples were collected for histology analysis. For tracking of *BMP*-hBMSC distribution in the newly form tissues, an eGFP antibody was used (Abcam) with the same IHC procedure described above.

### Micro-CT imaging

Ectopic bone formation, including morphology and mineralization, was monitored using micro-CT by means of a Scanco vivaCT 40 system (Wayne, PA) at 14, 28, and 56 days post-implantation. Before the imaging procedure, mice were anesthetized (3% isoflurane by inhalation for maintenance) and allowed to recover before returning to the housing room.

### Mechanical test

The mechanical properties of samples after 56 days of implantation were assessed using a material testing instrument (3200 Electroforce, Bose, MA) over a span of 8 mm at a cross-head displacement rate of 0.05 mm/second, as shown in Additional file 1: Figure S2A. Load-displacement curves were recorded, and peak forces were determined.

### Calcium quantitation

The samples were immersed in 1 ml of 1 N HCl and then ground into small pieces using a plastic pestle in 1.5-ml microcentrifuge tubes. After 3 days, undissolved tissues were pelleted by centrifugation at 12,000*g* for 10 min. The calcium concentration in the supernatant was then measured using a Calcium Colorimetric Assay Kit (Abnova, Taiwan).

### Histological analysis

After 56 days of intramuscular implantation, samples were fixed for 1 week and then decalcified for 1 h in a formic acid decalcifier (Immunocal, Decal Chemical Corporation, Tallman, NY). Decalcified samples were washed, dehydrated, embedded in paraffin and sectioned. Half of the samples were stained with hematoxylin and eosin (H&E) using a standard protocol. OCN IHC was performed as described before.

### Statistical analysis

Data are presented as the mean ± standard deviation (SD). Two-tailed Student’s *t* test was used for determining the statistical significance of two-group comparisons. At least three samples from each group were evaluated for all in vitro and in vivo experimental analyses.

## Results

### Efficiency of VL-PSL scaffold fabrication

As shown in Fig. 1a, VL-PSL was utilized to fabricate gelatin scaffolds that not only possessed a customized geometry and architecture but also containing encapsulated *BMP*-hBMSCs. The lentiviral *BMP-2* construct included a human *BMP-2* gene cassette followed by an *eGFP* reporter gene cassette. Both cassettes were driven by the CMV promoter, separately (Fig. 1b). *eGFP* gene expression allowed the detection of *BMP*-hBMSCs within the scaffolds and the assessment of gene transduction efficiency. Additional file 1: Figure S3 shows the same area under phase and fluorescence microscopy, showing lenti-*BMP-2*-transduced hBMSCs cultured on tissue culture plastic (TCP). The transduction efficiency was estimated to be approximately 65–70% at 1 week after transduction based on the ratio of eGFP-positive cells. No cytotoxicity was observed during the transduction process, as demonstrated by cell viability assessed by means of ethidium homodimer-1 (EthD-1) staining, as well as cell proliferation quantified using the CellTiter 96 Aqueous Cell Proliferation Assay on day 7 (data not shown).

### Activity of secreted BMP-2 and osteogenic gene expression of hBMSCs in 2D culture

The activity state of the BMP-2 produced by *BMP*-hBMSCs was evaluated by examining the level of osteogenesis stimulation in hBMSC cultures, based on histochemical assessment of ALP activity. As shown in Additional file 1: Figure S4A, *BMP*-hBMSCs showed high ALP activity, as indicated by strong blue staining, while naïve hBMSCs had no visible staining. The expression of genes related to osteogenesis, such as *OCN* and *BSP II*, in *BMP*-hBMSCs also showed a dramatic increase (Additional file 1: Figure S4B). Taken together, these results showed that the BMP-2 produced by *BMP*-hBMSCs was bioactive and able to drive hBMSCs efficiently toward osteogenic differentiation.

### Bone scaffold fabrication using VL-PSL

Constructs were next fabricated with *BMP*-hBMSCs incorporated into gelatin scaffolds using VL-PSL with different architectures (Additional file 1: Figure S1). Using our VL-PSL apparatus, the resolution could be as low as 70 μm, and the maximum dimension of scaffold was up to 90 mm × 75 mm × 100 mm (L × W × H). In this study, we used scaffolds with a cylindrical shape (5 mm D × 2 mm H), which could be manipulated readily for different tests and quantitative analyses. As control, constructs were also fabricated with naïve hBMSCs incorporated into gelatin scaffolds using VL-PSL, with the addition of BMP-2 protein (0.1 μg/ml). We used “Gene” and “Protein” to designate the scaffolds loaded with *BMP*-hBMSCs or naïve hBMSCs with BMP-2 protein, respectively. The morphology of infected hBMSCs within scaffold after VL-PSL was shown in Additional file 1: Figure S5.

Some of the 3D samples were cultured in growth medium for in vitro analysis, and the rest of samples were implanted into the muscle of severe combined immunodeficiency (SCID) mice for in vivo bone formation assessment.

### BMP-2 production and osteogenesis of hBMSCs within VL-PSL-fabricated constructs

**Fig. 2:**
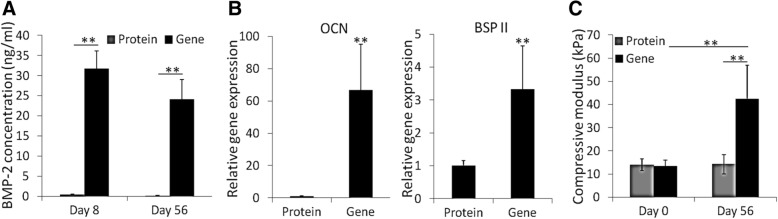
Bioactive BMP-2 produced by encapsulated *BMP*-hBMSCs promoted hBMSC osteogenesis. **a** BMP-2 concentration in the medium from the culture with the constructs from the Protein and Gene groups. Day 8 and Day 56 were the medium collection times. (*n* = 3). **b** Relative osteogenic gene expression in hBMSCs in the Protein and Gene groups. All data were normalized to the Protein group. (*n* = 3). **c** Compressive moduli of constructs from different groups at different time. (*n* = 4). **p < 0.05

After 3 days of in vitro culture, the VL-PSL fabricated constructs loaded with cells in the Gene or Protein group were placed in the fresh growth medium for another 5 days. The level of BMP-2 in the medium on day 8 and day 56, collected as medium in a 5-day culture period, was quantified using ELISA for BMP-2. As shown in Fig. 2a, after 5 days of culture, the BMP-2 concentration in the Gene group was 32 ng/ml, which was higher than the dose (10 ng/ml) reported to be required for the induction of hBMSC osteogenesis [48]. The BMP-2 concentration in the Protein group was < 0.5 ng/ml at the same time point, considerably lower than its initial concentration (100 ng/ml) or the concentration in the Gene group at same time point, as a result of release from the scaffold as well as dilution after the medium changes. As expected, significantly higher expression levels of the osteogenic genes, including *OCN* and *BSP II*, were detected in the hBMSCs in the Gene groups than those in the Protein group (Fig. 2b).

Interestingly, hBMSCs in the Protein group within the 3D scaffold had higher expression of *OCN* and *BSP II* than those grown on 2D tissue culture plastic (TCP, data not shown), suggesting that short exposure to BMP-2 and 3D gelatin scaffold culture was able to facilitate hBMSC osteogenesis to some extent. After 56 days of culture, the concentration of BMP-2 that accumulated for 5 days in the Gene group in the culture medium was measured to be 24 ng/ml, which was lower than that at the beginning but still higher than the effective concentration (10 ng/ml) needed for osteogenesis [48]. BMP-2 was not detectable in the Protein group at this time. Consistent with the observed levels of *BMP-2* gene expression, continuous *eGFP* gene expression was also observed in *BMP*-hBMSCs encapsulated within scaffolds. As shown in Additional file 1: Figure S6, strong eGFP expression remained detectable in the BMP-hBMSCs within the scaffolds at day 56.

**Fig. 3:**
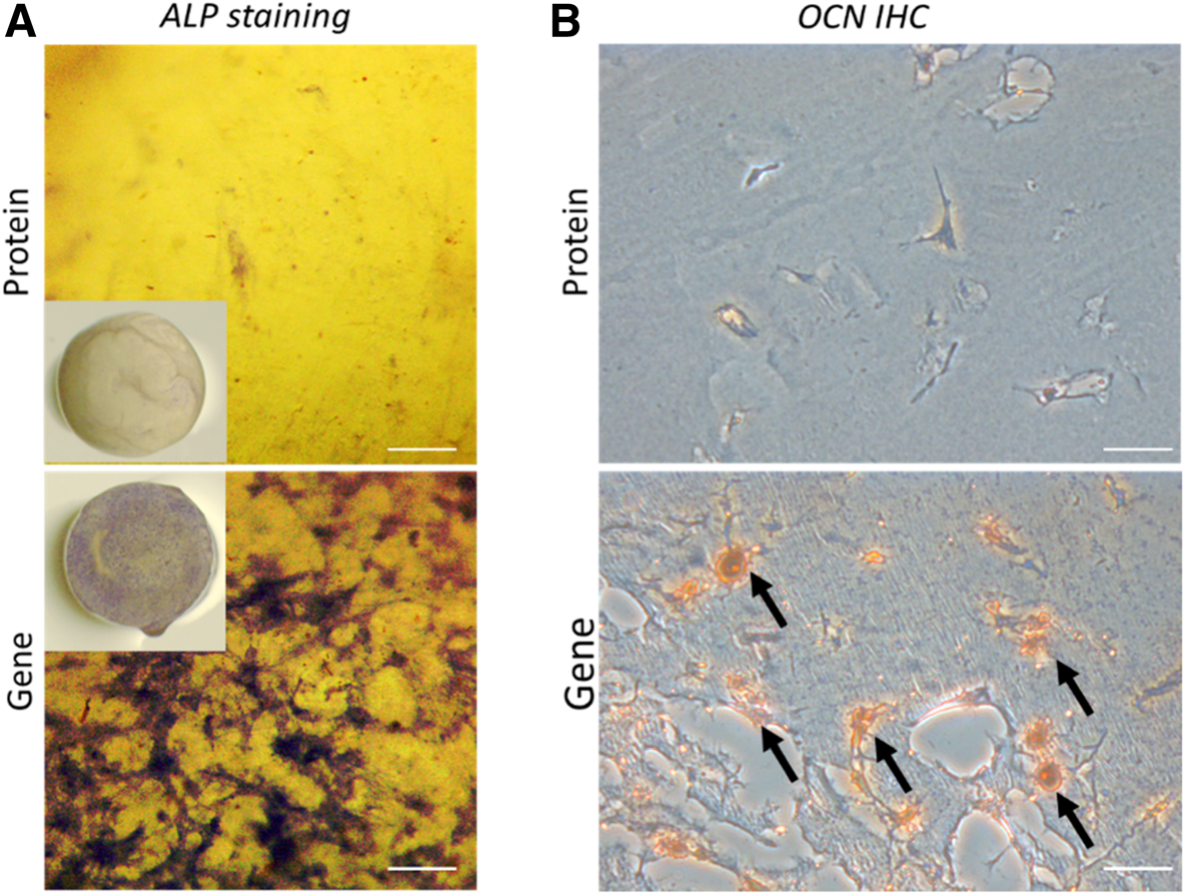
Scaffolds encapsulating *BMP*-hBMSCs showed much higher ALP activity and OCN deposition in the matrix. **a** ALP staining of the constructs in the Protein and Gene groups after 14 days of culture. The purple staining indicated the presence of ALP. **b** OCN deposition was detected by IHC in both groups after 14 days of culture. The brown staining, indicated by arrows, indicates OCN-positive deposition. Bar = 100 μm

In vitro osteogenesis was also assessed by ALP staining and OCN IHC. As shown in Fig. 3a, the hBMSCs in the Gene group showed substantially more intense staining than those in the Protein group, suggesting higher ALP expression. OCN IHC revealed OCN deposition in the constructs in the Gene group (Fig. 3b). In contrast, OCN-positive staining was sparse within the scaffolds in the Protein group (the control), indicating poor osteogenesis after 14 days of culture. Mechanical testing results provided further support. Immediately after fabrication, the constructs of the Gene and Protein groups had similar compressive modulus, with no significant difference (Fig. 2c). However, after 56 days of in vitro culture, the Gene group constructs were significantly stronger, attaining a compressive modulus of 42.5 kPa, compared to 13.4 kPa at the beginning of culture. In contrast, the stiffness of the constructs of the Protein group did not change substantially (14.2 kPa) and was significantly lower than that of the Gene group. In summary, hBMSCs that had been transduced with the *BMP-2* gene exhibited high level of BMP-2 production within the VL-PSL-fabricated gelatin scaffolds, and this high level of expression resulted in sustained osteogenesis of the encapsulated hBMSCs without the addition of exogenous BMP-2 protein.

### In vivo bone formation

**Fig. 4:**
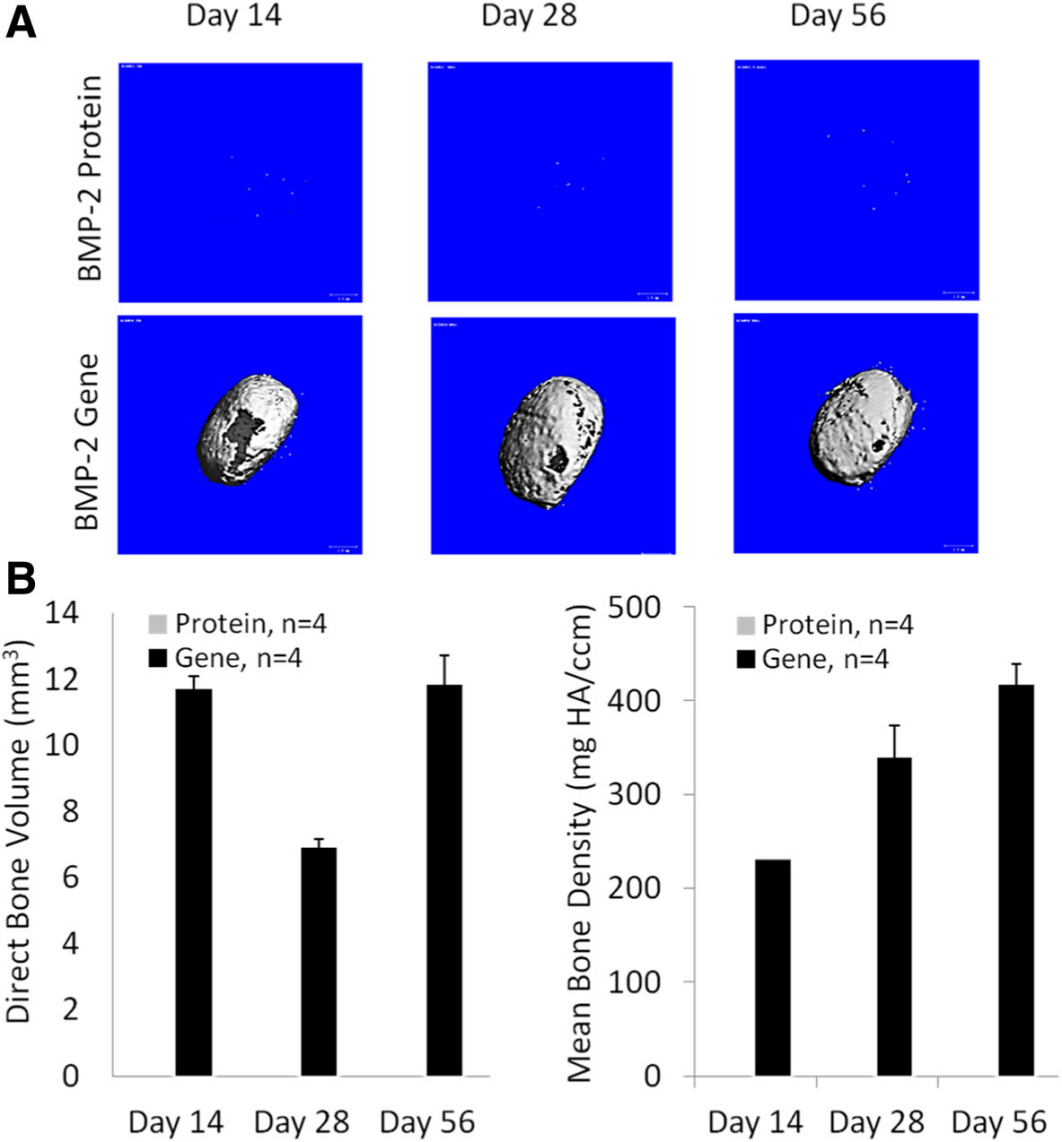
The micro-CT images and the bone volume and density of constructs at days 14, 28 and 56 days after implantation. **a** Representative reconstituted 3D micro-CT imaging from two groups at different time points. **b** Direct bone volume and mean bone density of new bone tissues in the Gene group at days 14, 28, and 56. (*n* = 4)

**Fig. 5:**
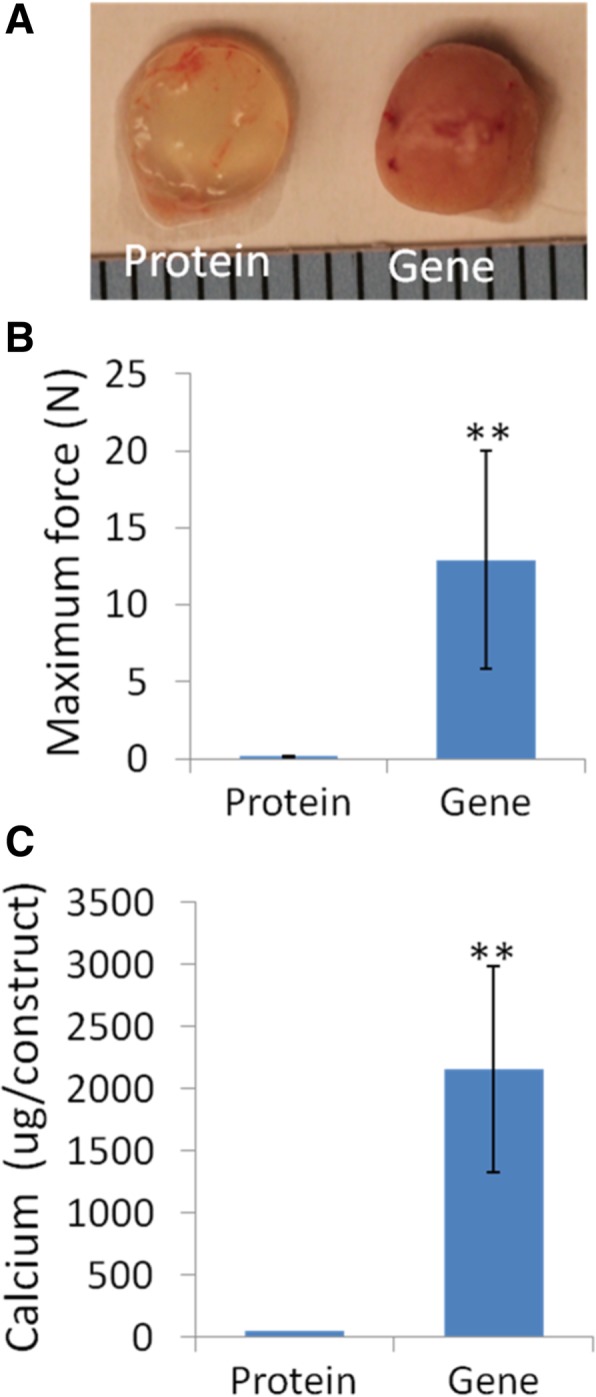
Constructs laden with lentiviral *BMP-2*-transduced hBMSCs were stiffer and had more calcium deposition after 56 days of implantation in vivo. **a** Macroscopic images showed new tissue formation in the Gene group construct. Scale = 1 mm, whereas those from the Protein Group remained morphologically unchanged. **b** Stiffness test. Maximum peak force was considerably higher in the Gene group constructs. (*n* = 3). **c** Total calcium content in the constructs. Again, the Gene group showed considerably higher calcium accumulation. (*n* = 3)

In vivo bone formation ability of the hBMSC-laden constructs was assessed by intramuscular implantation into SCID mice. Freshly fabricated constructs of the Gene or the Protein group were inserted into the hindlimb muscle pocket of SCID mice. At 14, 28, and 56 days post-operation, the mice were anesthetized and underwent micro-CT imaging of both implantation sites (Fig. 4a). Detectable mineralized areas in the *BMP*-hBMSC constructs were seen as early as day 14, suggesting rapid bone formation in the Gene group. With time, the new tissue became dense and more calcified. At day 56, the whole construct was completely ossified. Therefore, all samples were collected for analysis at this time point. In the control, consisting of similar implantation of a naïve cell/BMP-2 protein-laden construct, no sign of ossification was observed, which could be due to the low level of BMP-2 protein retained in the scaffolds, resulting from both the short half-life of BMP-2 and rapid systemic clearance by the bloodstream. As shown in Fig. 4b, the bone volume in the Gene group was 11.69 ± 0.41 mm^3^ at 14 days, subsequently reduced somewhat (6.89 ± 0.25 mm^3^ at 28 days), and then continuously increased to 11.8 ± 0.85 mm^3^ at 56 days. As expected, the bone density increased concomitantly and gradually with time, from 230.60 ± 0.28 to 338 ± 35.25 and then 417.12 ± 21.41 mg hydroxyapatite (HA)/ccm. Additionally, in comparison, as shown in Fig. 5a, the scaffolds of the Protein group maintained their structure and transparency after 56 days of in vivo implantation. In contrast, in the Gene group, the implanted constructs were no longer transparent. Importantly, the constructs maintained the cylindrical shape without irregular ossification outside, suggesting that most of the ossification happened within the constructs.

**Fig. 6:**
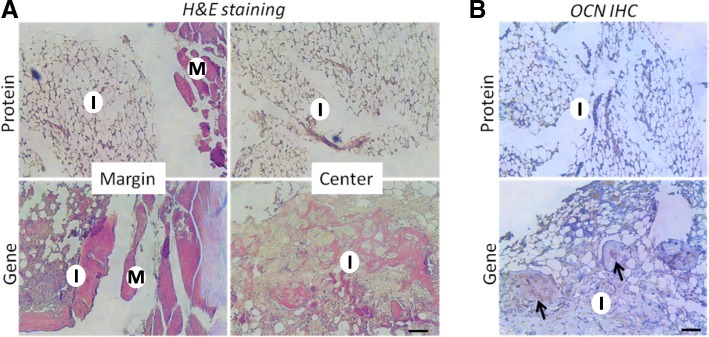
*BMP-2*-transduced hBMSCs promote bone formation in vivo after 56 days of implantation in vivo. **a** H&E staining. No bone tissue was observed in the Protein group; in contrast, in the Gene group, bone tissues were observed at the margin and in the center area, suggesting uniform bone formation throughout the scaffolds. **b** OCN IHC. Bone formation in the Gene group was further confirmed by strong OCN deposition (brown), indicated by arrows. Bar = 200 μm. I, implanted scaffolds; M, muscle tissue

The mechanical properties of the implants were then analyzed. Additional file 1: Figure S2 shows the test device (S2a) and typical force-displacement curves of the Gene and the Protein groups in this test (S2b). The highest force peaks were recorded (Fig. 5b). The Protein group showed much lower force than the Gene group. The results were consistent with the calcium quantitation data (Fig. 5c). The calcium density of the Gene group was approximately 40 μg/μl, which was higher than that in the mouse mandible bone (4.8 μg/μl) [49]. New bone formation after 56 days of implantation in vivo was further characterized by histological analysis. In the Protein group, only spongy structures were observed without bony tissue or any OCN-positive area (Fig. 6a and b**,** top panel). In the Gene group, new bone formation and OCN-positive staining were observed over a wide area from the edges to the center of the constructs. Many osteocytes were found in the interior of the new bone (Fig. 6a, b, bottom panel, arrows).

**Fig. 7:**
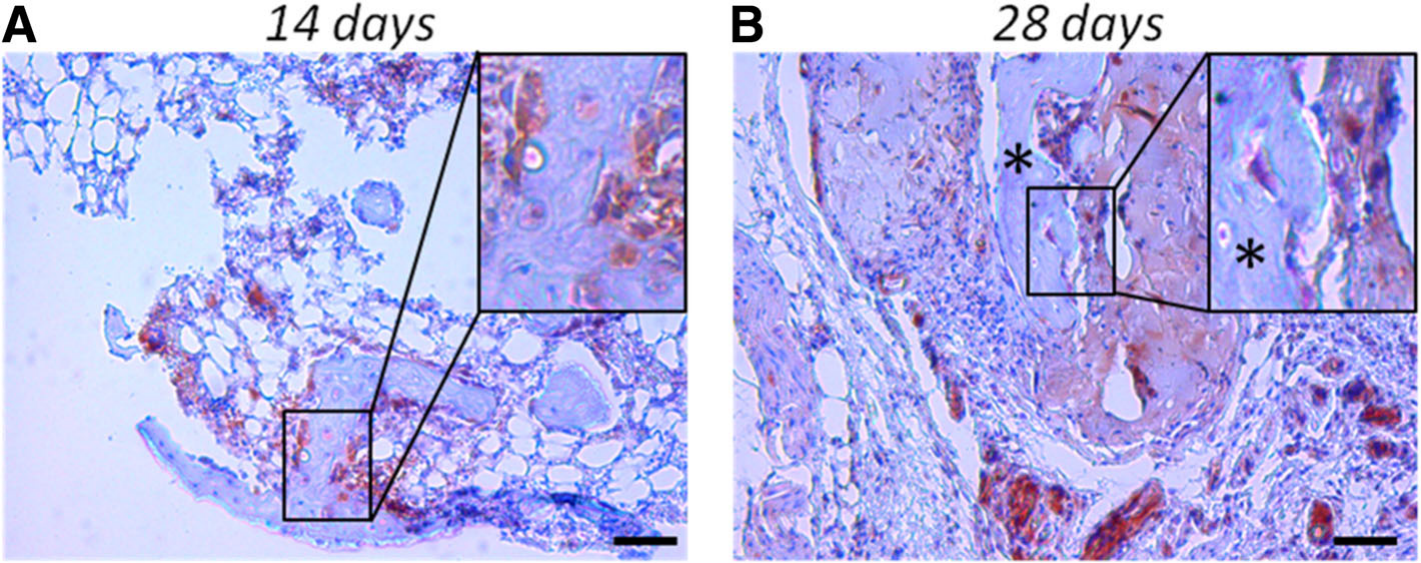
*BMP-2*-transduced hBMSCs were directly involved in new bone formation. GFP immunostaining was used to track the *BMP*-hBMSCs. **a** Fourteen days after implantation, massive engraftment of GFP-positive cells was seen not only on the edge of the new bone but also at the center. **b** At day 28, more bone tissue and increased GFP-positive area were observed. GFP-negative bone tissues (indicated by an asterisk) were also noticed, suggesting their origin from host cells. Bar = 200 μm

In a separate implantation study, we further investigated whether the grafted *BMP*-hBMSCs were directly involved in new bone formation beyond supplying BMP-2 by performing GFP IHC. As shown in Fig. 7a, *BMP*-hBMSCs were highly enriched in the early bone tissue area after 14 days. Most of these *BMP*-hBMSCs surrounded the bone tissues but some were clearly observed in the center, indicating the engraftment of hBMSCs. At day 28, the area of bone tissues increased, and GFP-positive regions were still seen throughout the tissues (Fig. 7b). We also noticed some GFP-negative bone tissues, which suggested that these tissues originated from host cells. In summary, *BMP*-hBMSCs not only produced BMP-2 to drive osteogenesis but also directly contributed to new bone formation by host cells.

## Discussion

Large bone defects and non-unions require bioactive constructs that not only match the defect anatomy structure but also drive sustained osteogenesis until healing is complete. In this study, we employed a novel VL-PSL technology to fabricate a structurally precise 3D bone scaffold using 3D models/medical imaging as the template and simultaneously incorporated hBMSCs that had been modified ex vivo with a lentiviral *BMP-2* construct to promote efficient osteogenesis within the cell-seeded scaffolds. The results showed efficient BMP-2 production in hBMSCs through the gene transfer approach that led to rapid and high-yield osteogenesis in vitro, as well as efficient ectopic bone formation uniformly throughout the construct upon intramuscular implantation in SCID mice.

The function of BMP-2 in promoting hBMSCs osteogenesis and bone regeneration has been widely tested in different animal models and clinical trials [50]. It has been reported that long-term BMP-2 activity within the defect site promotes more efficient bone formation than short-time activity [25, 51]. Therefore, stable, continuous, local delivery of bioactive BMP-2 to the defect site is critical for successful bone repair. Ex vivo gene transfer is an attractive potential option because this approach can result in BMP-2 production directly by the transplanted cells, overcoming the requirement of repeated BMP-2 admission or a complex protein delivery technology, as well as eliminating potential immune response caused by direct delivery of gene vectors in vivo [52]. For the purpose of cell tracking and transfection efficiency assessment, we have designed a lentiviral vector to co-express both *BMP-2* and *eGFP*, as demonstrated by the strong eGFP fluorescence signal in the *BMP*-hBMSCs within the construct for 56 days (Additional file 1: Figure S6).

Although applications of *BMP-2*-transduced hBMSCs for bone repair have been reported [39, 53], cell-based bone tissue engineering still needs to be further optimized. Due to the strong osteo-inductive capability of BMP-2, ex vivo transduced hBMSCs should not reach other healthy sites, especially at the early stage when high gene expression activity is high. Cell leakage from the defect site will also decrease effective cell number, thus impairing the regeneration effect. Therefore, a hydrogel scaffold able to retain the cells in position until the scaffolds degrade is desired. Among biodegradable biomaterials, gelatin scaffolds in different forms have been widely used because of their biocompatibility and because of their mechanical and biodegradable characteristics that represent a favorable microenvironment to promote biochemically, structurally matched natural bone healing [43]. On the basis of safety, gelatin has also been used as a carrier to deliver BMP-2 protein to a bone defect site [54]. Therefore, gelatin is a good vehicle for comparing the effects of the protein delivery and in vivo gene delivery strategy. As shown in Figs. 5 and 6, the scaffolds in the Gene group resulted in robust bone formation, as indicated by high bone density strictly found to be restricted within the constructs without outside growth, thus minimizing the side effects caused by bone formation outside of scaffolds. These results strongly suggest that the bone scaffolds used in this study maintain localized osteogenesis and bone formation within the construct, critical for the safety of tissue engineering products.

To achieve successful and long-term bone repair, structural and geometric matching of the biomaterial scaffold is another critical requirement. For example, a scaffold used for facial reconstitution or repair must retain the desired, original geometry in order to maintain human identity. Replication of the precise bone surface architecture after repair allows proper joint load distributions for normal daily activity [55]. Additionally, anatomic matching between grafts and host tissue will greatly reduce the gap between the graft and host tissues, and accelerate tissue fusion, critical for restoring the function of injured tissues. Therefore, the process of scaffold fabrication should ideally be capable of producing anatomically matched 3D scaffolds that fit the defect site, using computerized design or medical imaging, such as CT and magnetic resonance imaging (MRI) [56]. With conventional fabrication technology, it is very difficult or sometimes impossible to produce a patient- or defect-specific bone scaffold, despite the application of this technology in the engineering of a variety of tissues [57]. Among the various modeling methods, solid freeform fabrication (SFF) utilizes a computer to complete the whole process, thus promising the greatest ability to precisely control the geometry of bone scaffolds. For example, Shek et al. used an SFF polypropylene fumarate/TCP composite to fabricate a scaffold that could be matched to a specific patient defect geometry [58]. In the present study, we applied VL-PSL to produce a structurally precise, standard cylindrical shape to generate new bone constructs (Figs. 3, 4a, and 5a); in future studies, we will examine the application of medical imaging guided VL-PSL for other anatomically specific bone tissue printing.

Another significant advantage of this technology is that live cells were incorporated within the scaffolds, not only on the surface, which is often observed when cells are seeded onto scaffolds. Therefore, osteogenesis and matrix remodeling can take place throughout the entire structure of the scaffold, therefore not dependent on migration and/or invasion of cells from the surface to the inside. Such uniform cell distribution should dramatically reduce the time of complete new bone formation, especially within the depth of the scaffolds. As shown in micro-CT imaging and H&E staining (Figs. 4a and 6), bone formation (Gene group) in the center and margin area occur almost simultaneously, which is very important for uniform healing and proper bone function [24].

In this study, we have achieved approximately 70% transduction efficiency of *eGFP* gene in the hBMSCs, which is slightly lower than the efficiency observed in rabbit BMSCs (90%) [59], but the transduced hBMSCs produce significant quantities of BMP-2 up to 56 days, shown to be sufficient to promote long-term osteogenesis of hBMSCs, without prior cell sorting via flow cytometry. In comparison, the amount of BMP-2 found in the medium in the Protein group was very low due to early release from the scaffold and subsequent dilution by culture medium change. Another consideration is that the newly produced BMP-2 protein by the BMP-hBMSCs is physically bound to gelatin protein, thus resulting in its slow release, which has been exploited for BMP-2 delivery in another study [31]. Interestingly, the hBMSCs in the Protein group had enhanced osteogenic gene expression (Fig. 2b), indicating that the encapsulated BMP-2 had already initiated the osteogenesis process, but because subsequent BMP-2 levels were much lower in the Protein Group than the Gene group at the same time point, the hBMSCs in the Protein group were not able to form observable new bone. These results further support the importance of long-term BMP-2 activity in promoting bone formation [25].

The contribution of transplanted MSCs to the bone formation process has been reported [60, 61]. In our study, we observed BMP-hBMSCs localized within some, but not all, bone tissues (Fig. 7). This finding agreed with a previous report of a mixture of donor- and host-derived cells in regenerated bone [61]. It is noteworthy that a strongly eGFP-positive area was always present in the more naïve bone tissues than in mature bones, suggesting the critical role of *BMP*-hBMSCs during osteogenesis. This phenomenon has also been seen in other studies [60, 62]. This phenomenon is probably due to the replacement of donor cells by host cells or to a quiescent state of hBMSCs within the dense bone matrix, or more likely induced osteogenesis of host cells by the BMP-2 released by the BMP-hBMSCs.

There are some limitations in this study. For example, the photocrosslinked gelatin scaffold has relatively poor mechanical properties at the beginning (approximately 25 kPa compressive modulus), and the gel structure may not be optimal for bone formation. We have recently developed new biodegradable polymers made from poly-d,l-lactic acid/polyethylene glycol/poly-d,l-lactic acid (PDLLA-PEG), which had a compressive modulus up to 1.8 MPa [63, 64]. We are currently testing the utility of this novel biomaterial for bone tissue engineering. In addition, in this study, an ectopic bone formation model was used, which does not directly address the reparative potential of the methodology. The successful outcomes from this proof-of-concept study serve as the basis for future studies on direct bone repair in vivo, including the use of a larger animal model, such as rabbit.

## Conclusion

In this study, we have utilized VL-PSL to fabricate gelatin scaffolds laden with BMP-2-transduced hBMSCs, which showed long-term BMP-2 production (up to 56 days) and strongly promoted bone formation in vitro and in vivo. This *BMP-2*-expressing, hBMSC-encapsulating VL-PSL based construct is potentially applicable for the treatment of large bone defects. The medical imaging-guided fabrication capability of VL-PSL will further allow this bioactive scaffold to precisely fit the desired local anatomical structure. In further experiments, the repair capability of this bioactive construct in vivo will be determined by implantation in cranial or long bone defects.

## Additional file

Additional file 1: **Figure S1.** Under computerized control, VL-PSL is able to fabricate scaffolds with different geometries (A) and internal architectures, such as a porous structure (B). Bar = 5 mm in (B). **Figure S2.** Mechanical test. (A) The device for mechanical test. (B) Representative force-displacement curve of the tested samples from the Gene and Protein groups (in vivo study) in this test. **Figure S3.** hBMSC transduced with a lentiviral BMP-2 and eGFP containing gene construct and maintained in 2D culture. (A) Phase contrast microscopy; and (B) Epifluorescence microscopy. Bar = 200 μm. **Figure S4.** ALP activity and osteogenesis-associated gene expression in naïve hBMSCs (Control) and lentiviral BMP-2 construct transduced hBMSCs (BMP-2). The latter showed significantly higher ALP staining (A, purple) and enhanced *OCN* and *BSP II* expression as measured by real-time PCR (B). **Figure S5.** 3D confocal imaging of hBMSCs within scaffolds. hBMSCs (green) were infected with Lentiviral-BMP-2-eGFP. Bar = 100 μm. **Figure S6.** Strong eGFP expression in lentiviral BMP-2 transduced hBMSCs encapsulated in gelatin scaffolds remained after 56 days in culture. Bar = 100 μm. (PDF 1824 kb)

## Data Availability

The data sets supporting the conclusion of this article are included within the article and its additional files.

## Abbreviations

ALP: Alkaline phosphatase
BMP-2: Bone morphogenetic protein-2
BSP II: Bone sialoprotein II
eGFP: Enhanced green fluorescent protein
EthD-1: Ethidium homodimer-1
hBMSCs: Human bone marrow-derived stem cells
IHC: Immunohistochemistry
LAP: Lithium phenyl-2,4,6-trimethylbenzoyl phosphinate
mGL: Methacrylated gelatin
micro-CT: Microcomputed tomography
OCN: Osteocalcin
PEGDA: Poly (ethylene glycol) diacrylate
SCID: Severe combined immunodeficiency
VL-PSL: Visible-light-based projection stereolithography

## References

[1] Panteli M, Pountos I, Jones E, Giannoudis PV (2015). Biological and molecular profile of fracture non-union tissue: current insights. J Cell Mol Med.

[2] Munk B, Larsen CF (2004). Bone grafting the scaphoid nonunion - a systematic review of 147 publications including 5246 cases of scaphoid nonunion. Acta Orthop Scand.

[3] Kim DH, Vaccaro AR (2006). Osteoporotic compression fractures of the spine; current options and considerations for treatment. Spine J.

[4] Ewers R, Goriwoda W, Schopper C, Moser D, Spassova E (2004). Histologic findings at augmented bone areas supplied with two different bone substitute materials combined with sinus floor lifting. Report of one case. Clin Oral Implants Res.

[5] Shors EC (1999). Coralline bone graft substitutes. Orthop Clin North Am.

[6] Henkel J, Woodruff MA, Epari DR, Steck R, Glatt V, Dickinson IC (2013). Bone regeneration based on tissue engineering conceptions - a 21st century perspective. Bone Res.

[7] Langer R, Vacanti JP (1993). Tissue engineering. Science..

[8] Cancedda R, Dozin B, Giannoni P, Quarto R (2003). Tissue engineering and cell therapy of cartilage and bone. Matrix Biol.

[9] Hutmacher DW, Schantz JT, Lam CX, Tan KC, Lim TC (2007). State of the art and future directions of scaffold-based bone engineering from a biomaterials perspective. J Tissue Eng Regen Med.

[10] Tuli R, Nandi S, Li WJ, Tuli S, Huang X, Manner PA (2004). Human mesenchymal progenitor cell-based tissue engineering of a single-unit osteochondral construct. Tissue Eng.

[11] Stylios G, Wan T, Giannoudis P (2007). Present status and future potential of enhancing bone healing using nanotechnology. Injury.

[12] Tuan RS, Boland G, Tuli R (2003). Adult mesenchymal stem cells and cell-based tissue engineering. Arthritis Res Ther.

[13] Bruder SP, Kraus KH, Goldberg VM, Kadiyala S (1998). The effect of implants loaded with autologous mesenchymal stem cells on the healing of canine segmental bone defects. J Bone Joint Surg Am.

[14] Dallari D, Fini M, Stagni C, Torricelli P, Nicoli Aldini N, Giavaresi G (2006). In vivo study on the healing of bone defects treated with bone marrow stromal cells, platelet-rich plasma, and freeze-dried bone allografts, alone and in combination. J Orthop Res.

[15] Ito K, Yamada Y, Naiki T, Ueda M (2006). Simultaneous implant placement and bone regeneration around dental implants using tissue-engineered bone with fibrin glue, mesenchymal stem cells and platelet-rich plasma. Clin Oral Implants Res.

[16] Yuan J, Zhang WJ, Liu G, Wei M, Qi ZL, Liu W (2010). Repair of canine mandibular bone defects with bone marrow stromal cells and coral. Tissue Eng Part A..

[17] Zheng Y, Liu Y, Zhang CM, Zhang HY, Li WH, Shi S (2009). Stem cells from deciduous tooth repair mandibular defect in swine. J Dent Res.

[18] Kon E, Muraglia A, Corsi A, Bianco P, Marcacci M, Martin I (2000). Autologous bone marrow stromal cells loaded onto porous hydroxyapatite ceramic accelerate bone repair in critical-size defects of sheep long bones. J Biomed Mater Res.

[19] Berner A, Reichert JC, Woodruff MA, Saifzadeh S, Morris AJ, Epari DR (2013). Autologous vs. allogenic mesenchymal progenitor cells for the reconstruction of critical sized segmental tibial bone defects in aged sheep. Acta Biomater.

[20] Lee SH, Shin H (2007). Matrices and scaffolds for delivery of bioactive molecules in bone and cartilage tissue engineering. Adv Drug Deliv Rev.

[21] Einhorn TA, Majeska RJ, Mohaideen A, Kagel EM, Bouxsein ML, Turek TJ (2003). A single percutaneous injection of recombinant human bone morphogenetic protein-2 accelerates fracture repair. J Bone Joint Surg Am.

[22] Abbah SA, Lam WM, Hu T, Goh J, Wong HK (2013). Sequestration of rhBMP-2 into self-assembled polyelectrolyte complexes promotes anatomic localization of new bone in a porcine model of spinal reconstructive surgery. Tissue Eng Part A..

[23] Seo JP, Tsuzuki N, Haneda S, Yamada K, Furuoka H, Tabata Y (2014). Osteoinductivity of gelatin/beta-tricalcium phosphate sponges loaded with different concentrations of mesenchymal stem cells and bone morphogenetic protein-2 in an equine bone defect model. Vet Res Commun.

[24] Chen B, Lin H, Wang J, Zhao Y, Wang B, Zhao W (2007). Homogeneous osteogenesis and bone regeneration by demineralized bone matrix loading with collagen-targeting bone morphogenetic protein-2. Biomaterials..

[25] Jeon O, Song SJ, Yang HS, Bhang SH, Kang SW, Sung MA (2008). Long-term delivery enhances in vivo osteogenic efficacy of bone morphogenetic protein-2 compared to short-term delivery. Biochem Biophys Res Commun.

[26] Tabata Y (2006). Regenerative inductive therapy based on DDS technology of protein and gene. J Drug Target.

[27] Dmitriev AE, Lehman RA, Symes AJ (2011). Bone morphogenetic protein-2 and spinal arthrodesis: the basic science perspective on protein interaction with the nervous system. Spine J.

[28] Xia YJ, Xia H, Chen L, Ying QS, Yu X, Li LH (2018). Efficient delivery of recombinant human bone morphogenetic protein (rhBMP-2) with dextran sulfate-chitosan microspheres. Exp Ther Med.

[29] Hsiao HY, Yang SR, Brey EM, Chu IM, Cheng MH (2016). Hydrogel delivery of mesenchymal stem cell-expressing bone morphogenetic protein-2 enhances bone defect repair. Plast Reconstr Surg Glob Open.

[30] Bodde EW, Boerman OC, Russel FG, Mikos AG, Spauwen PH, Jansen JA (2008). The kinetic and biological activity of different loaded rhBMP-2 calcium phosphate cement implants in rats. J Biomed Mater Res A.

[31] Zhang Q, Tan K, Zhang Y, Ye Z, Tan WS, Lang M (2014). In situ controlled release of rhBMP-2 in gelatin-coated 3D porous poly (epsilon-caprolactone) scaffolds for homogeneous bone tissue formation. Biomacromolecules..

[32] Awad HA, Zhang X, Reynolds DG, Guldberg RE, O’Keefe RJ, Schwarz EM (2007). Recent advances in gene delivery for structural bone allografts. Tissue Eng.

[33] Chen Y, Luk KD, Cheung KM, Xu R, Lin MC, Lu WW (2003). Gene therapy for new bone formation using adeno-associated viral bone morphogenetic protein-2 vectors. Gene Ther.

[34] Lin H, Tang Y, Lozito TP, Oyster N, Kang RB, Fritch MR (2017). Projection stereolithographic fabrication of BMP-2 gene-activated matrix for bone tissue engineering. Sci Rep.

[35] Mi MT, Y; Salay, M; Li, G; Huard, J; Fu, F; Niyibizi, C; Wang, B. AAV based ex vivo gene therapy in rabbit adipose-derived mesenchymal stem/progenitor cells for osteogenesis. Open Stem Cell J. 2009;1:69–75.

[36] Kumar S, Mahendra G, Nagy TR, Ponnazhagan S (2004). Osteogenic differentiation of recombinant adeno-associated virus 2-transduced murine mesenchymal stem cells and development of an immunocompetent mouse model for ex vivo osteoporosis gene therapy. Hum Gene Ther.

[37] Martins AM, Santos MI, Azevedo HS, Malafaya PB, Reis RL (2008). Natural origin scaffolds with in situ pore forming capability for bone tissue engineering applications. Acta Biomater.

[38] Lin H, Zhang D, Alexander PG, Yang G, Tan J, Cheng AW (2013). Application of visible light-based projection stereolithography for live cell-scaffold fabrication with designed architecture. Biomaterials..

[39] Castro-Govea Y, Cervantes-Kardasch VH, Borrego-Soto G, Martinez-Rodriguez HG, Espinoza-Juarez M, Romero-Diaz V (2012). Human bone morphogenetic protein 2-transduced mesenchymal stem cells improve bone regeneration in a model of mandible distraction surgery. J Craniofac Surg..

[40] Chung VH, Chen AY, Jeng LB, Kwan CC, Cheng SH, Chang SC (2012). Engineered autologous bone marrow mesenchymal stem cells: alternative to cleft alveolar bone graft surgery. J Craniofac Surg.

[41] Yamada K, Tabata Y, Yamamoto K, Miyamoto S, Nagata I, Kikuchi H (1997). Potential efficacy of basic fibroblast growth factor incorporated in biodegradable hydrogels for skull bone regeneration. J Neurosurg.

[42] Tabata Y, Yamada K, Hong L, Miyamoto S, Hashimoto N, Ikada Y (1999). Skull bone regeneration in primates in response to basic fibroblast growth factor. J Neurosurg.

[43] Asamura S, Mochizuki Y, Yamamoto M, Tabata Y, Isogai N (2010). Bone regeneration using a bone morphogenetic protein-2 saturated slow-release gelatin hydrogel sheet: evaluation in a canine orbital floor fracture model. Ann Plast Surg.

[44] Fairbanks BD, Schwartz MP, Bowman CN, Anseth KS (2009). Photoinitiated polymerization of PEG-diacrylate with lithium phenyl-2,4,6-trimethylbenzoylphosphinate: polymerization rate and cytocompatibility. Biomaterials..

[45] Van den Bulcke AI, Bogdanov B, De Rooze N, Schacht EH, Cornelissen M, Berghmans H (2000). Structural and rheological properties of methacrylamide modified gelatin hydrogels. Biomacromolecules..

[46] Nichol JW, Koshy ST, Bae H, Hwang CM, Yamanlar S, Khademhosseini A (2010). Cell-laden microengineered gelatin methacrylate hydrogels. Biomaterials..

[47] Xiang G, Yang Q, Wang B, Sekiya N, Mu X, Tang Y (2011). Lentivirus-mediated Wnt11 gene transfer enhances Cardiomyogenic differentiation of skeletal muscle-derived stem cells. Mol Ther.

[48] Wang YK, Yu X, Cohen DM, Wozniak MA, Yang MT, Gao L (2012). Bone morphogenetic protein-2-induced signaling and osteogenesis is regulated by cell shape, RhoA/ROCK, and cytoskeletal tension. Stem Cells Dev.

[49] Seferos N, Kotsiou A, Petsaros S, Rallis G, Tesseromatis C (2010). Mandibular bone density and calcium content affected by different kind of stress in mice. J Musculoskelet Neuronal Interact.

[50] Lo KWH, Ulery BD, Ashe KM, Laurencin CT (2012). Studies of bone morphogenetic protein-based surgical repair. Adv Drug Deliv Rev.

[51] Brown KV, Li B, Guda T, Perrien DS, Guelcher SA, Wenke JC (2011). Improving bone formation in a rat femur segmental defect by controlling bone morphogenetic protein-2 release. Tissue Eng Part A.

[52] Nayak S, Herzog RW (2010). Progress and prospects: immune responses to viral vectors. Gene Ther.

[53] Chang SC, Chung HY, Tai CL, Chen PK, Lin TM, Jeng LB (2010). Repair of large cranial defects by hBMP-2 expressing bone marrow stromal cells: comparison between alginate and collagen type I systems. J Biomed Mater Res A.

[54] Patel ZS, Yamamoto M, Ueda H, Tabata Y, Mikos AG (2008). Biodegradable gelatin microparticles as delivery systems for the controlled release of bone morphogenetic protein-2. Acta Biomater.

[55] Ballyns JJ, Bonassar LJ (2009). Image-guided tissue engineering. J Cell Mol Med.

[56] Hollister SJ (2005). Porous scaffold design for tissue engineering. Nat Mater.

[57] Leong KF, Cheah CM, Chua CK (2003). Solid freeform fabrication of three-dimensional scaffolds for engineering replacement tissues and organs. Biomaterials..

[58] Schek RM, Taboas JM, Hollister SJ, Krebsbach PH (2005). Tissue engineering osteochondral implants for temporomandibular joint repair. Orthod Craniofac Res.

[59] Sugiyama O, An DS, Kung SP, Feeley BT, Gamradt S, Liu NQ (2005). Lentivirus-mediated gene transfer induces long-term transgene expression of BMP-2 in vitro and new bone formation in vivo. Mol Ther.

[60] Gao X, Usas A, Proto JD, Lu A, Cummins JH, Proctor A (2014). Role of donor and host cells in muscle-derived stem cell-mediated bone repair: differentiation vs. paracrine effects. FASEB J.

[61] Tatebe M, Nakamura R, Kagami H, Okada K, Ueda M (2005). Differentiation of transplanted mesenchymal stem cells in a large osteochondral defect in rabbit. Cytotherapy..

[62] Tortelli F, Tasso R, Loiacono F, Cancedda R (2010). The development of tissue-engineered bone of different origin through endochondral and intramembranous ossification following the implantation of mesenchymal stem cells and osteoblasts in a murine model. Biomaterials..

[63] Sun AX, Lin H, Fritch MR, Shen H, Alexander PG, DeHart M (2017). Chondrogenesis of human bone marrow mesenchymal stem cells in 3-dimensional, photocrosslinked hydrogel constructs: effect of cell seeding density and material stiffness. Acta Biomater.

[64] Sun AX, Lin H, Beck AM, Kilroy EJ, Tuan RS (2015). Projection stereolithographic fabrication of human adipose stem cell-incorporated biodegradable scaffolds for cartilage tissue engineering. Front Bioeng Biotechnol.

