# Supplementary material for: Efficient in vivo bone formation by BMP-2 engineered human mesenchymal stem cells encapsulated in a projection stereolithographically fabricated hydrogel scaffold
Source: Stem Cell Res Ther. 2019 Aug 14;10:254. doi: 10.1186/s13287-019-1350-6 (PMC6694509; doi:10.1186/s13287-019-1350-6)
Supplement: Supplementary file 1 — Figure S1. Under computerized control, VL-PSL is able to fabricate scaffolds with different geometries (A) and internal architectures, such as a porous structure (B). Bar = 5 mm in (B). Figure S2. Mechanical test. (A) The device for mechanical test. (B) Representative force-displacement curve of the tested samples from the Gene and Protein groups (in vivo study) in this test. Figure S3. hBMSC transduced with a lentiviral BMP-2 and eGFP containing gene construct and maintained in 2D culture. (A) Phase contrast microscopy; and (B) Epifluorescence microscopy. Bar = 200 μm. Figure S4. ALP activity and osteogenesis-associated gene expression in naïve hBMSCs (Control) and lentiviral BMP-2 construct transduced hBMSCs (BMP-2). The latter showed significantly higher ALP staining (A, purple) and enhanced OCN and BSP II expression as measured by real-time PCR (B). Figure S5. 3D confocal imaging of hBMSCs within scaffolds. hBMSCs (green) were infected with Lentiviral-BMP-2-eGFP. Bar = 100 μm. Figure S6. Strong eGFP expression in lentiviral BMP-2 transduced hBMSCs encapsulated in gelatin scaffolds remained after 56 days in culture. Bar = 100 μm. (PDF 1824 kb) [file 13287_2019_1350_MOESM1_ESM.pdf]

Supplemental Materials

Fig. S1

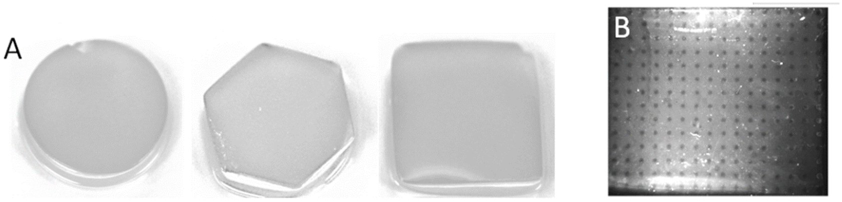

Fig. S1. Under computerized control, VL-PSL is able to fabricate scaffolds with different geometries (**A**) and internal architectures, such as a porous structure (**B**). Bar = 5 mm in **(B)**.

Fig. S2

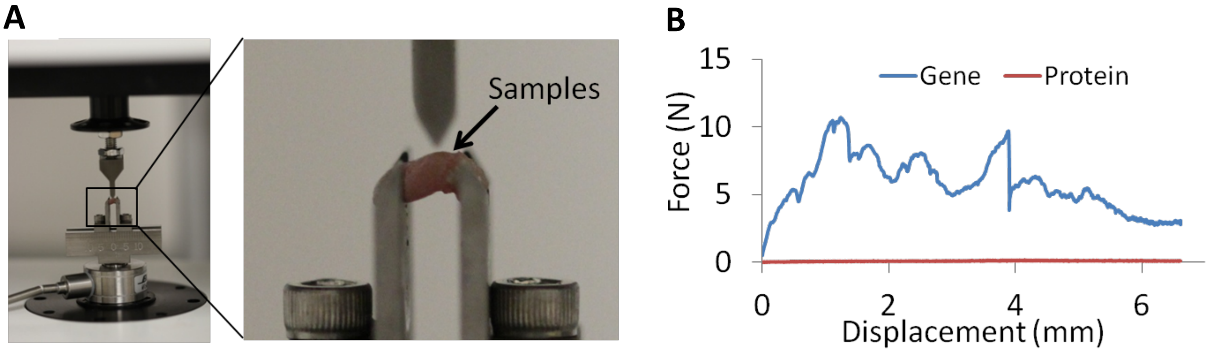

Fig. S2. Mechanical test. **(A)** The device for mechanical test. **(B)** Representative force-displacement curve of the tested samples from the Gene and Protein groups (*in vivo* study) in this test.

Fig. S3

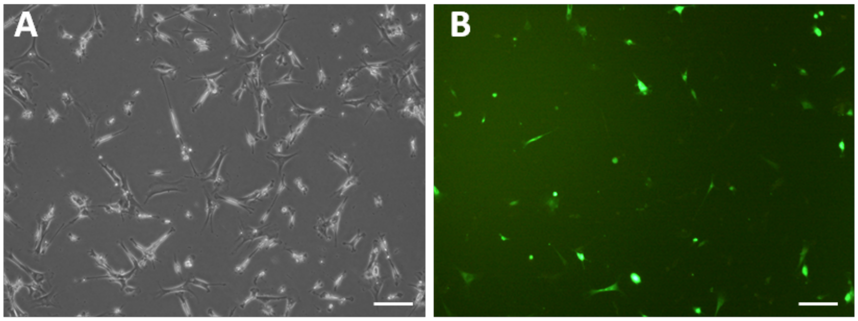

Fig. S3. hBMSC transduced with a lentiviral BMP-2 and eGFP containing gene construct and maintained in 2D culture. **(A)** Phase contrast microscopy; and **(B)** Epifluorescence microscopy. Bar = 200 μm.

Fig. S4

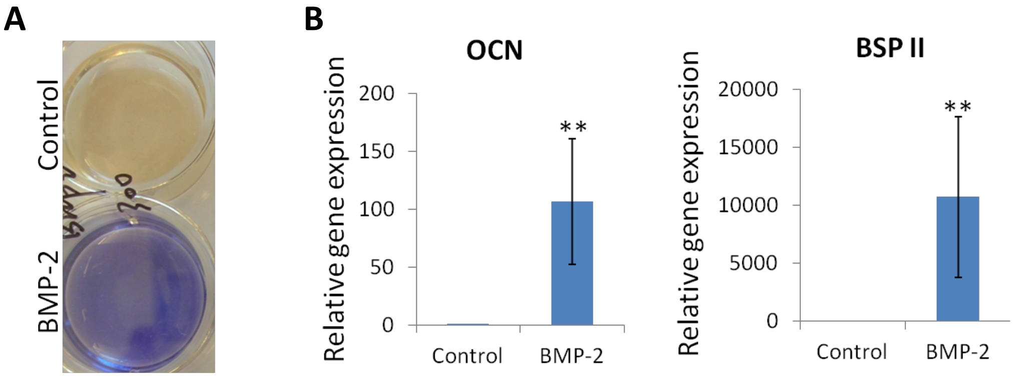

Fig. S4. ALP activity and osteogenesis-associated gene expression in naïve hBMSCs (Control) and lentiviral BMP-2 construct transduced hBMSCs (BMP-2). The latter showed significantly higher ALP staining (**A**, purple) and enhanced *OCN* and *BSP II* expression as measured by real-time PCR (**B**).

Fig. S5

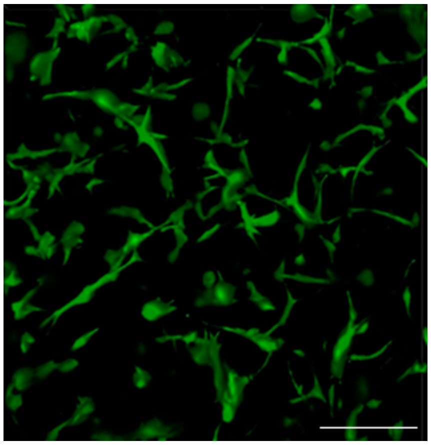

Fig. S5. 3D confocal imaging of hBMSCs within scaffolds. hBMSCs (green) were infected with Lentiviral-BMP-2-eGFP. Bar = 100 µm.

Fig. S6

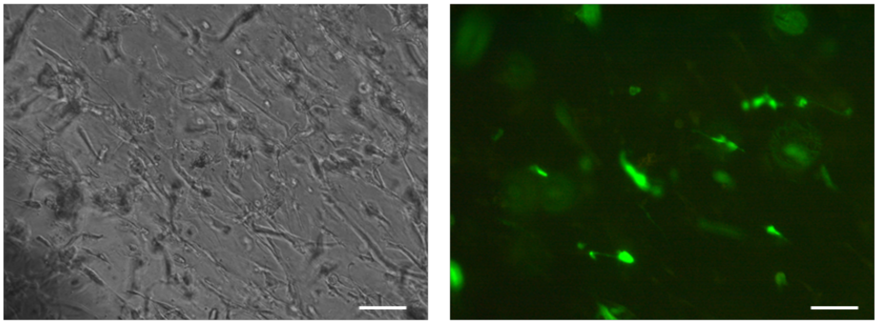

Fig. S6. Strong eGFP expression in lentiviral BMP-2 transduced hBMSCs encapsulated in gelatin scaffolds remained after 56 days in culture. Bar = 100 µm.
